# NGF in Neuropathic Pain: Understanding Its Role and Therapeutic Opportunities

**DOI:** 10.3390/cimb47020093

**Published:** 2025-01-31

**Authors:** Mario García-Domínguez

**Affiliations:** 1 Program of Immunology and Immunotherapy, CIMA-Universidad de Navarra, 31008 Pamplona, Spain; mgdom@unav.es; 2 Department of Immunology and Immunotherapy, Clínica Universidad de Navarra, 31008 Pamplona, Spain; 3 Centro de Investigación Biomédica en Red de Cáncer (CIBERONC), 28029 Madrid, Spain

**Keywords:** nerve growth factor, neuropathic pain, neurotrophin, analgesia

## Abstract

Nerve growth factor (NGF) is one of the essential components that have been implicated in the pathophysiology of neuropathic pain, a condition that develops following nerve injury or dysfunction. This neurotrophin is critical for the survival and maintenance of sensory neurons, and its dysregulation has been implicated in the sensitization of pain pathways. NGF interacts with its receptor TrkA and p75^NTR^ to activate intracellular signaling pathways associated with nociception and the emergence of allodynia and hyperalgesia. Therapeutic approaches employing neutralizing antibodies and molecule inhibitors have been highly effective at both preclinical and clinical levels, hence giving hope again for the use of NGF as an important biomarker and therapeutic target in the management of neuropathic pain. By exploiting the unique properties of NGF and its interactions within the nervous system, new therapeutic modalities could be designed to enhance efficacy while minimizing side effects. In conclusion, taking advantage of the multifaceted dynamics of NGF could provide effective pain management therapies to finally respond to the unmet needs of patients experiencing neuropathic pain.

## 1. Introduction

The International Association for the Study of Pain (IASP) has defined pain as an “unpleasant sensory and emotional experience associated with, or resembling that associated with, actual or potential tissue damage” [1,2]. Pain may be differentiated into several types, depending on a variety of characteristics, such as origin [3]: nociceptive pain, which results from the activation of nociceptors (as a result of potential or real damage to non-neural tissues); nociplastic pain, originating from altered nociception (resulting from a disease or lesion of the somatosensory system, with no clear evidence of actual or potential tissue damage); and neuropathic pain, due to a clear disease or lesion in the somatosensory nervous system.

Neuropathic pain can be caused by a direct injury to the nervous system [4]. Symptoms for neuropathic pain are described by patients through characteristic sensations like burning and cold, electric feelings, tingling and needles, numbness, and hypersensitivity [5]. These symptoms may be associated with allodynia and hyperalgesia [6]. The underlying mechanisms of neuropathic pain involve both peripheral (PNS) and central nervous system (CNS) alterations [7,8,9]. Peripheral sensitization encompasses hyperexcitability of injured nerve fibers, increased spontaneous activity, and hypersensitivity to mechanical and thermal stimuli [8]. Central sensitization refers to the amplification of neural transmission and processing through the spinal cord and brain, resulting in amplified pain signaling [9].

Neuropathic pain can result from some conditions [4,5]: diabetic neuropathy (DPN), chemotherapy-induced peripheral neuropathy (CIPN), spinal cord injury, postherpetic neuralgia, trigeminal neuralgia, multiple sclerosis, and HIV (human immunodeficiency virus)-associated neuropathy. Prevalence rates have been variable in many epidemiological studies, reflecting major methodological differences in the assessment methods and heterogeneous study populations. Nevertheless, recent large-scale studies give more reliable estimates. In particular, the estimated overall prevalence of neuropathic pain was given to be 9.2% in the UK Biobank [10]. This is further supported by additional estimates, which place the prevalence between 6.9% and 10% in the general population [11]. Reported prevalence rates for Spain and other European countries range from 6% to 8% [12,13]. 

Neuropathic pain results from the multifaceted action of several molecules influencing the onset and maintenance of the disease. These include ion channels, which are critical in pain signaling, such as voltage-gated sodium channels (VGSCs), especially Nav1.7, Nav1.8, and Nav1.9 [14], and the α_2_δ subunit of voltage-gated calcium channels (VGCCs) [15]. It is also essential to mention the TRP (transient receptor potential) channels, particularly TRPA1 and TRPV1, which are associated with thermal and mechanical hypersensitivity, as well as peripheral sensitization [16]. On the other hand, pro-inflammatory cytokines (e.g., TNF-α, IL-1β, and IL-6) and chemokines (e.g., CCL2 and CX3CL1) promote central sensitization, along with neurotrophic factors such as NGF (nerve growth factor) and BDNF (brain-derived neurotrophic factor) [17]. 

Neurotrophic factors play a crucial role in the development and maintenance of neuropathic pain, influencing neuronal survival, growth, and plasticity [18]. NGF plays a fundamental role in the development and maintenance of neuropathic pain by affecting neuronal survival, growth, and plasticity [19]. In neuropathic conditions, NGF is expressed at elevated levels both in the dorsal root ganglion (DRG) and the spinal dorsal horn, leading to sensitization and enhancement of pain signaling [20,21]. NGF binds to the TrkA (tropomyosin receptor kinase A) receptor, activating pathways that boost neuronal excitability and upregulate the expression of VGSCs, thereby promoting central sensitization [22]. 

Neuropathic pain is complex and requires an integrated approach involving pharmacological interventions (e.g., tricyclic antidepressants (TCAs), serotonin–norepinephrine reuptake inhibitors (SNRIs), opioids, and gabapentinoids) and interventional techniques such as nerve blocks, spinal cord stimulation, and intrathecal drug delivery systems [23,24]. However, with the growing body of research on neuropathic pain, new therapeutic targets are being explored, such as the use of anti-TNF-α antibodies [25], anti-IL-20 [26], anti-MMP9 (matrix metalloproteinase 9) [27], and anti-NGF [28].

This review will detail the fundamental characteristics of NGF and key aspects of neuropathic pain, including its etiology, underlying mechanisms, clinical presentation, and impact on the quality of life of affected patients. Additionally, this work aims to compile research on the application of NGF antagonists in the treatment of neuropathic pain. In conclusion, this review seeks to enhance the reader’s understanding of how NGF treatments can manage neuropathic pain.

## 2. Characteristics of NGF

NGF, which was discovered in 1956 by Rita Levi-Montalcini and Stanley Cohen [29], is a neurotrophic factor that enhances the growth and survival of peripheral sensory and sympathetic nerve cells in mammals, including humans [30]. NGF was the first member identified in the neurotrophin family, which includes BDNF, neurotrophin 3 (NT-3), and neurotrophin 4 (NT-4) [30].

NGF acts through two receptors: the tropomyosin receptor kinase A (TrkA), which has tyrosine kinase activity, and the p75 neurotrophin receptor (p75^NTR^), known as the ‘high-affinity receptor” and “low-affinity receptor”, respectively [31]. Initial observations of NGF date back to 1949 when Rita Levi-Montalcini, using malignant mouse tumor fragments, induced the invasion of sensory fibers in chick embryos and hypothesized that such tissues release a growth-stimulating agent [32]. By the beginning of the 1950s, Rita Levi-Montalcini and Stanley Cohen performed numerous experiments that characterized this agent, including the purification and identification of NGF as a protein in 1960 [33]. Several studies have emphasized the crucial role that NGF plays in various biological processes. NGF is not only important during development and for the maintenance of sympathetic and sensory ganglia [34], but it also acts on a wide range of cell types. Its actions extend to the PNS and the CNS, influencing neural growth, survival, and plasticity [35,36]. Moreover, NGF has been implicated in modulating immune responses and maintaining homeostasis, further underscoring the multifaceted role of NGF in neuroimmune regulation and general biological balance [37,38].

### 2.1. Structure

**Figure 1:**
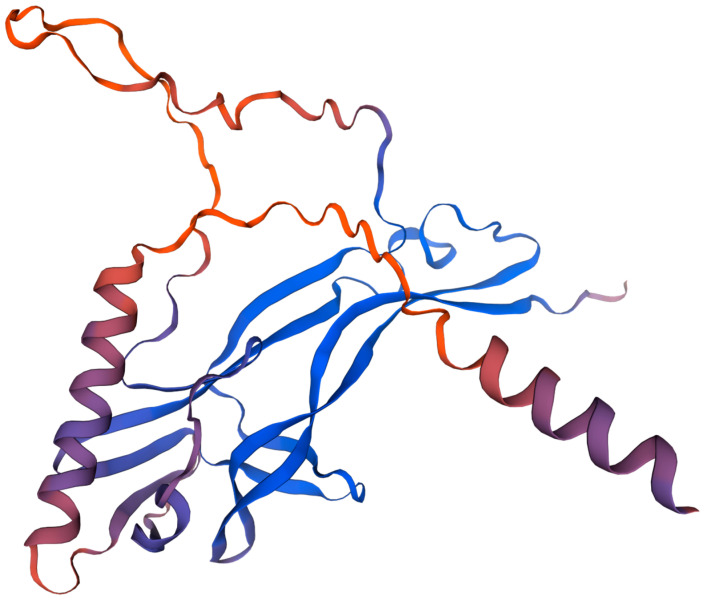
Tridimensional structure of human NGF. Image generated using Expasy software 3.0.

The structure of NGF is characterized by its complexity (Figure 1) and was first defined in 1991 via X-ray crystallography [39]. NGF is a glycoprotein composed of a homodimer with two identical polypeptide chains [40], showing an obligate parallel dimer, with each of the protomers forming a β sandwich. Each protomer is composed of 118 amino acids, which fold into a distinctive three-dimensional conformation, featuring four distinct loop regions and two beta-pleated strands [41]. 

The dimeric form of NGF, with a molecular weight of 12.5 kDa per monomer, is stabilized by hydrophobic interactions and hydrogen bonds [42]. These loops, which are rich in polar and charged amino acids, are oriented toward the external environment, providing NGF with unique structural characteristics relative to other neurotrophins [42]. In contrast, the β-strands are located at the dimer interface, contributing to the structural similarity with other members of the neurotrophin family [43].

Each monomer is further stabilized by a cysteine knot motif formed by three disulfide bridges, which play a crucial role in maintaining structural integrity and biological functions [44]. The structure of the NGF dimer assumes a butterfly-like conformation, with its monomers constituting the wings. This conformation permits it to bind its receptors, the TrkA and p75^NTR^, facilitating neuronal survival and differentiation through signaling cascades. Binding sites for these receptors are found in opposite parts of the dimer, allowing simultaneous engagement with more than one receptor and the capability to form higher-order complexes for signal transduction [45]. These findings are supported by small-angle X-ray scattering (SAXS) and nuclear magnetic resonance (NMR) studies [46,47]. SAXS provides useful information regarding the general shape and size of a macromolecule in solution, in which researchers can identify structural features at low resolution that reflect how these molecules behave in more physiologically relevant environments [48]. On the other hand, NMR facilitates the investigation of key functional aspects of biomolecules in their natural conditions [49].

### 2.2. Biosynthesis and Degradation

The human *Ngf* gene is situated on the short arm of chromosome 1 (1p13.2) and spans 52 kb, consisting of three exons interrupted by two introns [50]. NGF mRNA is processed through alternative splicing, producing multiple transcript variants that encode two different protein isoforms [51]. These variants are vital for the diverse functions of NGF in different tissues and developmental stages.

The biosynthesis of NGF is a complex process involving multiple enzymatic steps and cellular compartments. NGF is initially synthesized as a precursor protein, proNGF, with a molecular weight of approximately 32 kDa. This precursor is produced in the rough endoplasmic reticulum, where the signal peptide is cleaved by signal peptidase. Subsequently, proNGF undergoes N-linked glycosylation in the rough endoplasmic reticulum and Golgi apparatus, facilitated by putative transferases, but the exact glycosylation sites and pattern have never been investigated thoroughly [52,53,54]. ProNGF undergoes post-translational modifications, including the formation of disulfide bonds (mediated by protein disulfide isomerases) [55,56], as well as proteolytic cleavage that occurs both intracellularly and extracellularly. Intracellular cleavage is mediated by furin, a proprotein convertase found in the trans-Golgi network [57], and extracellular processing can be carried out by tissue plasminogen activator (tPA) and plasmin [58,59]. Once NGF has exerted its biological effects, it is degraded by MMP9 [60]. The MMP9 enzyme belongs to a large family involved in the degradation of components of the extracellular matrix and in the regulation of several cellular events [61]. NGF degradation by MMP9 regulates its availability and activity within the cellular environment to prevent its excessive signaling effect [60].

The regulation of NGF expression is very complex at both the transcriptional and translational levels. NGF is transcriptionally regulated by numerous transcription factors, which include Sp1 (specificity protein 1) and AP-1 (activating protein-1) binding to certain promoter regions in the *Ngf* gene [62,63]. These proteins can be activated under different stimuli, such as oxidative stress or mechanical trauma, leading to enhanced *Ngf* gene expression [64,65]. In addition, some drugs, like valproic acid, have been shown to enhance NGF transcription [66]. Other neurotrophic factors and signaling cascades take part in NGF expression regulation. So far, the involvement of BDNF in NGF expression has been suggested, highlighting the complex interactions between different neurotrophic factors [67]. 

### 2.3. Distribution and Biological Functions

Neurotrophins can interact with two distinct types of receptors, each with a different structural family [68]: tropomyosin receptor kinases (known as Trks) and a member of the tumor necrosis factor receptor (TNFR) superfamily (known as p75^NTR^). Specifically, NGF interacts with TrkA [69] and p75^NTR^ [70]. 

**Figure 2:**
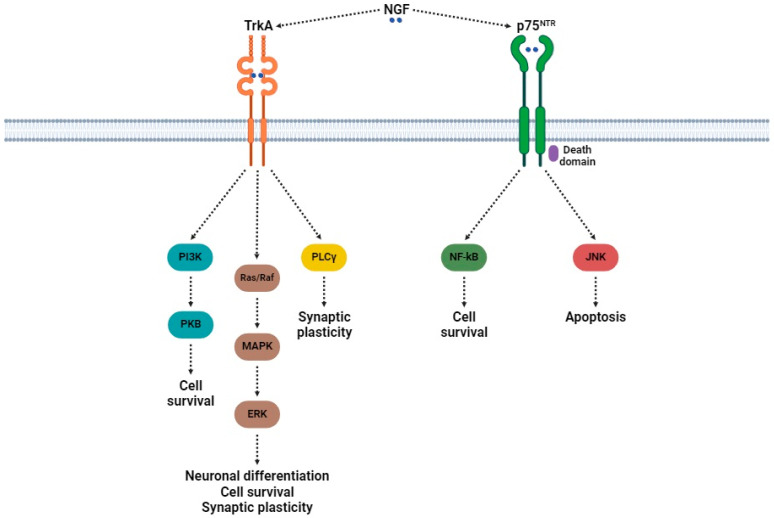
Signaling pathways mediated by TrkA and p75^NTR^, receptors of NGF. Abbreviations: NGF (nerve growth factor); TrkA (tropomyosin receptor kinase A); p75^NTR^ (p75 neurotrophin receptor); PI3K (phosphoinositide 3-kinase); PKB (protein kinase B); Ras (rat sarcoma virus); Raf (rapidly accelerated fibrosarcoma); MAPK (mitogen-activated protein kinase); ERK (extracellular signal-regulated kinase); PLCγ (phospholipase C gamma); NF-κB (nuclear factor kappa-light-chain-enhancer of activated B cells); JNK (c-Jun N-terminal kinase).

TrkA is a 140 kDa transmembrane glycoprotein (796 amino acids) that exhibits a high affinity for NGF and belongs to the receptor tropomyosin kinase family. This receptor is encoded by the *Ntrk1* gene, which is situated on chromosome 1p23.1 [71]. This receptor is classified as a type I transmembrane protein composed of extracellular domains (ECDs), which include two cysteine-rich motifs (CRMs (domains 1 and 3)) separated by three leucine-rich motifs (LRMs (domain 2)), followed by two immunoglobulin-like motifs (domains 4 and 5) established in the juxtamembrane region. Domain 5 is responsible for binding NGF [72]. TrkA signaling (Figure 2) is important for neuronal survival, growth, and differentiation. Upon NGF binding, TrkA receptors dimerize and undergo autophosphorylation of some residues, which activates the principal downstream pathways involving phospholipase C gamma (PLCγ), mitogen-activated protein kinase (MAPK)/extracellular signal-regulated kinase (ERK), and phosphoinositide 3-kinase (PI3K)/protein kinase B (PKB; also known as Akt) [73,74]. While TrkA signaling is initiated at the axon terminal, further support (through signaling endosomes that are transported retrogradely to the cell body) allows NGF to influence gene expression [75]. Its activity is modulated by its co-receptor (p75^NTR^), which can enhance or suppress the function of TrkA.

The p75^NTR^ receptor, which has a very low affinity for NGF, is also classified as a type I transmembrane receptor. Its extracellular domain contains four repeated modules, each consisting of six cysteines. Each cysteine-rich domain (CRD1-CRD4) forms three disulfide bridges that contribute to the structural integrity of the receptor [45]. All the cysteine-rich repeats participate in binding to NGF [40]. There are two variants of the p75^NTR^ receptor: the full-length receptor and an alternatively spliced isoform that excludes exon III, which contains CRD2-CRD4 [45]. The full-length form is cleaved by a metalloproteinase to produce an ECD, which binds NGF and releases a free-floating extracellular domain [45]. This receptor exhibits a complex function in neuronal signaling, affecting both survival and apoptotic pathways. The p75^NTR^ receptor serves as a co-receptor for TrkA, which enhances its activity, promoting survival and growth [76]. On the other hand, p75^NTR^ independently mediates other signaling pathways that can activate either apoptosis or survival, depending on the cellular context. The p75^NTR^ receptor is associated with some adaptor proteins, including NRAGE (Maged1, dlxin), NADE (p75^NTR^-associated cell death executor), and NRIF (neurotrophin receptor-interacting factor), all of which are fully linked to apoptosis [77]. Other adaptor proteins, like FAP-1 (Fas-associated phosphatase-1), RIP2 (receptor-interacting protein 2), and TRAF6 (tumor necrosis factor receptor-associated factor 6) are associated with survival [77]. Upon activation of TrkA, p75^NTR^ is cleaved by α- and γ-secretase, generating an intracellular domain referred to as p75^ICD^. This domain is crucial for PKB phosphorylation after neurotrophin treatment and in some processes controlled by TrkA [78]. 

NGF has been found to be widely distributed across the CNS. The highest levels of NGF mRNA are found in the cortex and hippocampus, suggesting that NGF acts as a trophic factor for cholinergic neurons placed in these CNS regions [79,80]. High levels of NGF mRNA are also found in regions containing the central processes of NGF-responsive sensory neurons, including the pons, medulla, and spinal cord [81]. The bioavailability of NGF mRNA in these areas is particularly significant, as it suggests that NGF supports and sustains NGF-responsive sensory neurons that project to these regions [81]. The striatum also contains NGF mRNA, indicating that NGF might function as a trophic factor for a population of NGF-responsive cholinergic local circuit neurons [82]. The distribution of NGF within the CNS undergoes alterations throughout development. In the cerebellum of developing macaques, NGF receptor immunoreactivity is present on Purkinje cells, granule cells of the premigratory zone of the external granule layer, and neurons of the deep nuclei but becomes diminished during development [83]. The presence of NGF in multiple regions of the CNS further supports the notion that NGF functions as a target-derived trophic factor for various neuronal populations, playing an essential role in the survival and development of CNS neurons [83]. NGF has a wide distribution in the PNS, where it plays critical roles in neuronal development and survival. In the autonomic nervous system, all sympathetic neurons but only some parasympathetic and enteric neurons exhibit detectable levels of p75^NTR^ [84]. 

NGF is found throughout peripheral tissues as well as in neural tissues. NGF (within the skin) is synthetized by keratinocytes [85], fibroblasts [86], and mast cells [87]. This production contributes to the survival and function of sensory neurons innervating the epidermis and is essential for wound healing and tissue repair processes in the skin [85,86]. Salivary glands, especially submandibular glands, are a rich source of NGF, contributing to oral wound healing [88]. In the reproductive system, NGF is present in the prostate, influencing glandular morphogenesis and secretory function [89]. On the other hand, NGF is present in the ovaries, where it takes part in follicular development and ovulation [90]. 

Another important site of NGF distribution is the immune system. Among the immune cells known to store and release NGF are mast cells, involved in neurogenic inflammation and allergic responses [87,91]. By releasing NGF, these cells facilitate communication between the nervous and immune systems, thereby influencing various processes such as pain signaling, tissue repair, and the modulation of inflammatory responses [21]. Of particular interest, NGF released by mast cells is thought to enhance neurogenic inflammation (a process in which nerve cells themselves further amplify allergic reactions and hypersensitivity by contributing to the inflammatory response) [92]. 

In the cardiovascular system, both cardiomyocytes and vascular smooth muscle cells produce NGF, influencing cardiac innervation and vascular tone [93]. NGF is present in the gastrointestinal tract and participates in gut motility and controls secretion [94]. In the respiratory system, NGF is highly expressed in the lungs and airways, where it has been shown to actively participate in neurogenic inflammation and bronchial hyperresponsiveness associated with diseases such as asthma [95].

## 3. Neuropathic Pain: A Comprehensive Overview

Neuropathic pain is a complex and chronic condition that directly results from injury or disease affecting the somatic nervous system (SNS), which is responsible for processing sensory information [4,5]. This pain can originate from peripheral and central sources, depending on whether the damage occurs in the PNS or the CNS [7,8,9]. Patients often refer to neuropathic pain manifestations such as burning sensations, tingling, or electric shocks. These sensory experiences are frequently accompanied by an increased sensitivity to light touch (known as allodynia), which indicates that a stimulus that is normally non-painful may lead to significant discomfort. Moreover, patients may manifest a powerful response to painful stimuli (known as hyperalgesia), even at minor levels of discomfort that could feel intolerable [6].

Neuropathic pain prevalence estimates range from 6.9% to 10%, thus underscoring the importance of this condition in public health [11]. Although significant progress has been made in understanding the neurobiological mechanisms underlying this disease, its management continues to be highly challenging. The primary form of treatment is pharmacological; however, some symptoms can be alleviated through interventional procedures to enhance patients’ quality of life.

### 3.1. Molecular Basis of Neuropathic Pain

**Figure 3:**
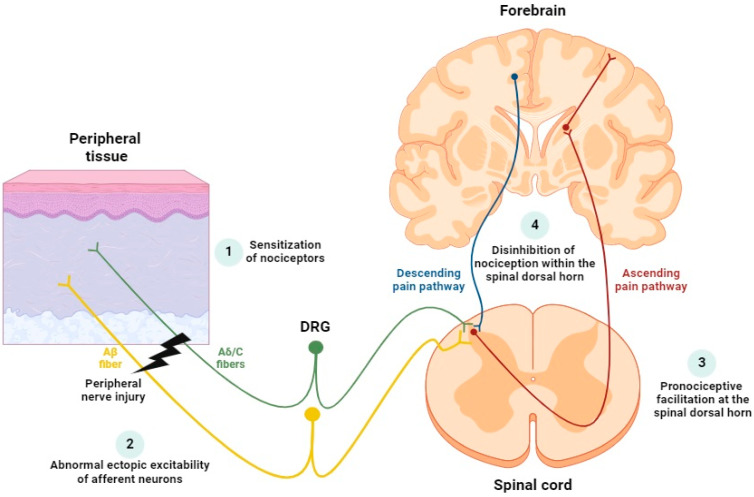
Cellular mechanisms that occur following damage to a peripheral nerve: (1) sensitization of nociceptors; (2) abnormal ectopic excitability of afferent neurons; (3) pronociceptive facilitation at the spinal dorsal horn; (4) disinhibition of nociception within the spinal dorsal horn. Abbreviation: DRG (dorsal root ganglion).

In recent years, many mechanisms that perpetuate neuropathic pain have been clarified (Figure 3):

1. Sensitization of nociceptors: Nociceptors are located at the free nerve endings of unmyelinated C and lightly myelinated Aδ fibers, which constitute the peripheral sensitization in neuropathic pain [96,97]. The action of nociceptors can be triggered by several agents, including inflammatory mediators (such as bradykinin, prostaglandins, neurokinins, calcitonin gene-related peptide (CGRP)) [98] and growth factors (NGF and BDNF) [18].

After peripheral nerve injury, immune cell infiltration and release of pro-inflammatory cytokines (such as IL-1β, IL-6, and TNF-α) lead to the development of allodynia and hyperalgesia [96,97]. Changes also occur in intracellular signaling pathways, including second messengers such as cAMP and NO, and numerous types of protein kinases (PKA, PKB, PKC, and MAPK) [99,100].

2. Abnormal ectopic excitability of afferent neurons: Spontaneous discharges from myelinated Aβ fibers produce paraesthesias and dysesthesias, while altered excitability of myelinated Aδ and unmyelinated C fibers produces burning pain [101,102]. These symptoms have been strongly associated with unusual activity of VGSCs (e.g., Nav1.7, Nav1.8, and Nav1.9) [103], among others.
3. Pronociceptive facilitation at the spinal cord level: Symptoms such as pin-prick hyperalgesia, cold hyperalgesia, and dynamic allodynia are indicative of central sensitization [104]. The transmission of pain signals involves ionotropic glutamate receptors [105,106], such as AMPA (α-amino-3-hydroxy-5-methyl-4-isoxazolepropionic acid) and NMDA (N-methyl-D-aspartate), as well as metabotropic glutamate receptors (mGluRs).

AMPA receptors are responsible for responses to acute pain [107], while NMDA receptors, normally under the inhibitory influence of Mg²⁺, amplify noxious input in conditions of sustained depolarization [108,109]. The activation of mGluRs modulates pain by enhancing synaptic transmission [110]. Central sensitization initiates a cascade of events leading to changes in intracellular signaling pathways, activating mechanisms such as PKC, MAPK, and NO synthetase, leading to long-term potentiation (LTP) and synaptic plasticity [111,112,113,114]. This process increases the excitability of central neurons, making them sensitive even to low-level inputs from C, Aδ, and Aβ fibers. As a result, neurons that usually require a higher threshold for activation can respond to weaker stimuli that would usually go undetected [115].

4. Disinhibition of nociception within the spinal dorsal horn: One of the major factors underlying the development and maintenance of neuropathic pain is the disinhibition of nociception in the spinal cord [116]. This occurs under conditions where the inhibitory mechanisms that normally dampen the nociceptive signals, such as GABAergic and glycinergic neurotransmission, are impaired [117]. Disinhibition involves the loss of inhibitory interneurons, disruptions in chloride balance, and altered receptor function, with synaptic plasticity ultimately sustaining persistent hyperexcitability in the spinal cord [118,119]. This hyperexcitability can have profound implications for neural communication and contribute highly to many neurological conditions [120].

### 3.2. Role of NGF in Neuropathic Pain

NGF levels are usually increased in some preclinical neuropathic pain models (Table 1). NGF has an impact on the pathogenesis of neuropathic pain through its effects on both peripheral and central pain pathways [121]. NGF primarily binds to its high-affinity receptor (TrkA), established in nociceptors, triggering downstream signaling cascades and enhancing neuronal excitability while promoting the expression of ion channels, crucial for pain signal transmission [122]. However, NGF induces the release of pro-inflammatory mediators, which potentiate pain signaling and enhance the inflammatory milieu [21]. 

This fact indicates that NGF is involved in peripheral sensitization, as the NGF-TrkA complex is internalized and retrogradely transported to the sensory neuron cell bodies (placed in the DRG), where it induces transcriptional changes that lead to a phenotypic alteration of the neurons [75]. This results in an increased expression of pain-related ion channels and receptors, including VGSCs [123], VGCCs [124], TRPs (such as TRPV1) [125], and acid-sensing ion channels (ASICs) [126]. The increased expression of these ion channels reduces the activation threshold of the nociceptors, thus making them more sensitive to noxious stimuli [127]. Additionally, NGF increases the production of neuropeptides such as substance P and CGRP from sensory nerve endings to cause neurogenic inflammation and further sensitization [128]. Another effect of this retrograde signaling is the elevated expression of BDNF in DRG neurons. This process is very important for neuronal plasticity, including connectivity and activity changes in neurons based on various experiences or environmental modifications. It allows the nervous system to respond properly in cases of injury or constant pain by stimulating changes at the molecular and cellular levels [129]. 

On the other hand, NGF induces the sprouting of sympathetic neurons that result in the abnormal connection between sympathetic and sensory fibers, which may result in sympathetically maintained pain [130]. Sensitization produced by NGF is not limited to direct neuronal actions but also encompasses interactions with immune cells and fibroblast-like synovial cells [38]. Moreover, NGF evokes mast cell degranulation and enhances the production of pro-inflammatory cytokines (e.g., TNF-α, IL-1β, and IL-6), which further acts in a positive feedback manner to amplify and perpetuate sensitization [38]. On the other hand, high levels of NGF in the PNS trigger the activation of p75^NTR^, which can interact with the high-affinity NGF receptor TrkA to modulate nociceptive signaling [131]. In this regard, NGF-induced mechanical hyperalgesia by intradermal injection of NGF into the rat hind paw was mediated by the p75^NTR^ signaling cascade, suggesting its involvement in pain modulation [132]. Posterior studies showed that suppression of p75^NTR^ in intact sensory neurons reduced neuropathic pain after nerve injury, indicating the importance of this receptor in maintaining pain [133]. The interaction of p75^NTR^ with NGF also modulates the expression of pain-related ion channels, including TRPV1, further contributing to neuronal hypersensitivity [132,134]. Surprisingly, investigation of the NGF R100W mutation, responsible for the onset and development of hereditary sensory and autonomic neuropathy type V (HSAN V), revealed that NGF signaling works synergistically to drive neuroplastic changes in peripheral nociceptors [135,136]. 

**Table 1:** List of conditions that associate the presence of NGF with the onset and maintenance of neuropathic pain, derived from both preclinical models and human clinical trials. Abbreviations: NGF (nerve growth factor); DRG (dorsal root ganglion); CCI (chronic constriction injury); SNI (spared nerve injury); RN (red nucleus); SCN (sciatic nerve cryoneurolysis); TN (trigeminal neuralgia); NP (neuropathic pain); OA (osteoarthritis); CNS (central nervous system); EAE (experimental autoimmune encephalomyelitis); CSF (cerebrospinal fluid); MS (multiple sclerosis); OA (osteoarthritis); DPN (diabetic polyneuropathy); CGRP (calcitonin gene-related peptide); HIV (human immunodeficiency virus); CIPN (chemotherapy-induced peripheral neuropathy); TrkA (tropomyosin receptor kinase A); TRPV1 (transient receptor potential vanilloid 1).

Cause of Neuropathy	Preclinical/Clinical Research	Role of NGF	References
Chronic constriction injury (CCI) model	Preclinical (rat)	The exogenous administration of NGF has led to a significant decrease in paw withdrawal latency times, highlighting the critical role of NGF in the development of hyperalgesia	[137]
	Preclinical (rat)	mRNA encoding NGF was present in cells at the site of injury and in the DRG at the lesion’s level. Also, NGF was significantly higher in the ganglia on the ipsilateral side of the CCI	[138]
	Preclinical (mouse)	Exogenous NGF exacerbated both mechanical and thermal allodynia induced by CCI. High levels of endogenous NGF also promoted sprouting within the DRG	[139]
	Preclinical (rat)	NGF contents were augmented within the spinal cord and the DRG following CCI. This increase in NGF contributed to the long-term reduction in tactile and mechanical thresholds after injury	[140]
	Preclinical (rat)	NGF expression was increased in the DRG and sciatic nerve of CCI rats	[141]
Spared nerve injury (SNI) model	Preclinical (rat)	The NGF levels in the red nucleus (RN) of SNI rats were significantly elevated compared to those of sham-operated rats	[142]
	Preclinical (mouse)	mRNA NGF levels increased in the injured DRG	[143]
Sciatica model induced by intervertebral disc herniation	Preclinical (rat)	This study ablated joint afferents by using the neurotoxin saporin conjugated to a ligand targeted to neurons involved in either peptidergic signaling and investigated the contributions of those neuronal populations to facet-mediated pain. The neurotoxin saporin prevented NGF-induced mechanical and thermal hypersensitivity in the forepaws	[144]
Peripheral nerve injury model (transection of lumbar spinal nerve)	Preclinical (rat)	Results confirmed that NGF played a significant role in the development of allodynia following a nerve injury	[145]
Sciatic nerve cryoneurolysis (SCN) model	Preclinical (rat)	Increased levels of NGF were found in the spinal dorsal horn of SCN rats manifesting hyperalgesia	[146]
Trigeminal neuralgia (TN)	Preclinical (rat)	Increased NGF levels were found in the ipsilateral infraorbital nerve branch at the time point corresponding to the peak of heat hyperalgesia	[147]
Multiple sclerosis (MS)	Preclinical (rat)	This study revealed that activated glial cells overexpress NGF mRNA in the CNS of EAE-affected rats. This suggests that elevated NGF levels in EAE rats’ brains are generated by glial cells	[148]
	Clinical	NGF was increased in the CSF of MS patients with central NP	[149]
Osteoarthritis (OA)	Clinical	NGF expression was induced in chondrocytes by mechanical and inflammatory stimuli	[150]
	Preclinical (mouse)	NGF expression was increased in the DRG of mice with osteoarthritis	[151]
	Preclinical (rat)	During osteoarthritis progression, NGF expression varied by tissue and disease stage. NGF increased in the synovium while continuing to rise in osteochondral channels and bone marrow. This suggests that NGF was a key driver of nerve growth linked to OA pain	[152]
Diabetic polyneuropathy (DPN)	Clinical	These studies have reported significant dose-dependent hyperalgesia at the site of NGF injection	[153,154]
	Preclinical (rat)	The pronociceptive role of NGF in diabetic rats was evidenced by the increased concentrations of CGRP and substance P found in both the DRG and the spinal dorsal horn	[155]
	Preclinical (mouse)	This study hypothesized that NGF participates in the development of mechanical allodynia by enhancing the expression of substance P and CGRP. Indeed, an increase in the expression of NGF, substance P, and CGRP genes at the onset of mechanical allodynia has been demonstrated in the DRG of db/db mice	[156]
HIV-associated neuropathy	Clinical	These studies have reported significant dose-dependent hyperalgesia at the site of NGF injection	[157,158]
Chemotherapy-induced peripheral neuropathy (CIPN)	Clinical	Serum NGF levels were elevated in cancer patients with painful CIPN receiving either taxane or platinum. Also, NGF may act as a biomarker of the presence and severity of NP in these populations	[159]
	Preclinical (rat)	NGF promoted sensory neuritogenesis and sensitized nociceptors. This effect was blocked by the TrkA antagonist GW441756. The administration of this antagonist inhibited TRPV1-mediated nociceptor sensitization induced by cisplatin, thereby preventing the onset of NP associated with this chemotherapeutic agent	[160]

Within the CNS, NGF plays a fundamental role in synaptic plasticity and neuronal excitability, thereby amplifying pain signaling [21]. In the spinal cord, NGF promotes the secretion of many pro-inflammatory mediators and excitatory neurotransmitters, such as glutamate, CGRP, and substance P, thereby enhancing nociceptive transmission [140,161,162]. Moreover, NGF signaling integrates with other important pathways, including the BDNF-TrkB pathway, which may amplify its role in central sensitization [163]. The role of NGF in central sensitization is not restricted to the spinal cord alone but extends supraspinally, for example, to the anterior cingulate cortex [140]. 

These findings, the role of NGF in both peripheral and central sensitization, its interaction with the immune system, and its involvement in promoting nerve fiber sprouting, represent notable progress in pain research. NGF has emerged as a key player in the development and maintenance of neuropathic pain, a process that has driven the need for continued efforts toward the elucidation and development of new therapies. This advancement promises a comprehensive approach to pain management in the future, tailored to individual patient profiles and the specific factors driving their pain [19]. 

## 4. Treatments Against NGF in Neuropathic Pain

Considering that neuropathic pain is often complex and typically associated with nerve damage or dysfunction, NGF appears to be a promising target for its treatment (Table 2). This protein has been strongly implicated in the development and maintenance of neuropathic pain, and its influence on peripheral and central sensitization processes is documented [164]. 

Anti-NGF treatments (Table 2) have emerged as promising strategies for managing neuropathic pain, offering new hope for a patient population that generally finds conventional therapies largely ineffective. These therapies include antibodies against NGF, which target a crucial mediator of chronic painful conditions, with neuropathic pain occupying an important place. In fact, many studies have already given evidence of anti-NGF therapies being effective in reducing pain sensitivity in different neuropathic pain models, such as chronic constriction injury [138,140,141,165,166,167,168,169], trigeminal neuralgia [147], osteoarthritis [172,173,174,175,176], DPN [177,178,179], and CIPN [160,180]. Indeed, the efficacy of many anti-NGF therapies surpasses merely immediate analgesic effects; studies suggest long-lasting benefits. The anti-NGF treatments have shown success in some inflammatory pain models, although efficacy in peripheral neuropathic conditions might be more limited, and further studies are needed to define their applicability in the clinical realm [181]. Despite all the challenges during clinical development, anti-NGF therapies remain under investigation as a remarkable innovation in chronic pain management, offering immense potential to enhance the overall quality of life for patients with neuropathic pain. 

Alternatively, NGF inhibitors (Table 2) can be considered an emerging class of therapeutic agents in the treatment of neuropathic pain. These represent a targeted approach as compared to traditional analgesics. NGF inhibitors remain under investigation as treatments for some forms of neuropathic pain, including osteoarthritis [174], CIPN [160], and chronic low back pain (LBP) [170]. Currently, numerous NGF inhibitors are under active investigation for neuropathic pain, with efforts toward increasing efficacy, minimizing side effects, and evaluating the most appropriate patient populations.

## 5. Conclusions

NGF has emerged as a critical factor in the pathophysiology of neuropathic pain, a condition that typically arises from abnormalities in pain perception due to injury or dysfunction of the nervous system. NGF inhibitors, which include numerous monoclonal antibodies and antagonists, have been developed to disrupt this interaction and reduce pain signals.

Indeed, many clinical studies have identified significant improvements in pain severity, functional outcomes, and overall quality of life in response to these NGF-targeted therapies. This suggests a therapeutic benefit associated with these agents compared to traditional analgesics, which are usually linked to undesirable side effects and generally limited efficacy. Despite the promising results, further research is essential to gain a deeper understanding of their long-term safety, mechanisms of action, and predictive biomarkers related to treatment responses. This insight will help optimize therapeutic strategies and ensure that these interventions are both effective and safe for patients over extended periods [182]. 

Finally, NGF represents a promising frontier in the management of neuropathic pain, offering new hope for patients who have previously experienced limited responses to various treatment modalities. Additional studies and clinical trials will be necessary to develop these treatments, determine their optimal application in clinical practice, and enhance our understanding of the role these factors play in pain modulation [182]. 

## Figures and Tables

**Table 2:** Compilation of numerous anti-NGF drug treatments employed for different neuropathic pain conditions. Abbreviations: CCI (chronic constriction injury); NGF (nerve growth factor); pAb (polyclonal antibody); mAb (monoclonal antibody); TrkA (tropomyosin receptor kinase A); IgG (immunoglobulin G); NP (neuropathic pain); MAPKK (mitogen-activated protein kinase kinase); MEK1/2 (mitogen-activated protein kinase/extracellular signal-regulated kinase kinase 1/2); ERK1/2 (extracellular signal-regulated kinase 1/2); TAK1 (TGF-β-activated kinase 1); MAPK (mitogen-activated protein kinase); p65 (transcription factor p65); *l*-CDL (levo-corydalmine); NF-κB (nuclear factor kappa-light-chain-enhancer of activated B cells); LBP (chronic low back pain); JNK (c-Jun N-terminal kinase); SNI (spared nerve injury); RN (red nucleus); DRG (dorsal root ganglion); TN (trigeminal neuralgia); OA (osteoarthritis); DPN (diabetic polyneuropathy); CIPN (chemotherapy-induced peripheral neuropathy); TRPV1 (transient receptor potential vanilloid 1).

Cause of Neuropathy	Preclinical/Clinical Research	Treatment Employed	Beneficial Results	References
Chronic constriction injury (CCI) model	Preclinical (rat)	Anti-NGF pAb	Inhibition of collateral sprouting by the saphenous nerve into the sciatic nerve’s territory was effectively prevented by the local application of anti-NGF	[165]
	Preclinical (rat)	Anti-NGF pAb	The application of anti-NGF serum at the injury site delayed the onset of hyperalgesia	[138]
	Preclinical (rat)	Anti-NGF mAb	High dosage of anti-NGF completely abolished heat and cold hyperalgesia, induced by CCI	[166]
	Preclinical (rat)	TrkA-IgG (inhibitor that comprises the NGF receptor linked to an immunoglobulin)	Inhibition of NGF after peripheral nerve injury reduced neuroma formation and NP while safeguarding the cell bodies of transected neurons	[167]
	Preclinical (rat)	PD90859 (inhibitor of the MAPKK family members MEK1/2 and blocks NGF-induced ERK1/2 phosphorylation)	PD98059 reduced pain scores and increased the effectiveness of opioids in neuropathy	[168]
	Preclinical (rat)	Anti-NGF mAb	Anti-NGF induced a significant, dose-dependent reduction in mechanical threshold, thermal withdrawal latency, and cold sensitivity	[140]
	Preclinical (rat)	Anti-NGF mAb *l*-CDL (inhibitor of NGF secretion)	Anti-NGF suppressed TAK1 in the periphery, reducing CCI-induced NP by inhibiting downstream MAPK and p65 signaling. Additionally, *l*-CDL inhibited NGF secretion by macrophages and Schwann cells, as well as downstream TAK1-MAPK/NF-κB signaling in the periphery, to alleviate CCI-induced NP	[141]
	Preclinical (mouse)	Y1036 (NGF sequestration agent)	Y1036 prevented NP-induced pain hypersensitivity	[169]
Chronic low back pain (LBP)	Preclinical (rat)	SP600125 (JNK inhibitor)	SP600125 reduced astrocyte and neuronal activation, demonstrating that the hypersensitivity and anxiety-like behaviors induced by NGF in LBP rats can be mitigated by this JNK inhibitor	[170]
Spared nerve injury (SNI) model	Preclinical (rat)	Anti-NGF mAb	Anti-NGF antibody was injected into the RN. The anti-NGF antibody attenuated mechanical allodynia	[142]
Peripheral nerve injury model (transection of lumbar spinal nerve)	Preclinical (rat)	ALE-0540 (TrkA antagonist)	Administration of ALE-0540 in rats resulted in antiallodynic effects in the L5/L6 spinal nerve ligation model	[171]
	Preclinical (rat)	Anti-NGF mAb	Direct delivery of anti-NGF antibodies into the injured DRG reduced the percentage of foot withdrawal responses	[145]
Trigeminal neuralgia (TN)	Preclinical (rat)	Anti-NGF mAb	Treatment with anti-NGF significantly alleviated heat hyperalgesia linked to trigeminal neuralgia	[147]
Osteoarthritis (OA)	Human	Anti-NGF mAb (Fulranumab)	Primary efficacy results showed that fulranumab significantly reduced the average pain intensity score	[172]
	Preclinical (rat)	Anti-NGF mAb	Anti-NGF mAb exerted a long-lasting analgesic effect	[173]
	Preclinical (rat)	AR786 (selective TrkA antagonist)	AR786 treatment prevented the development of pain behaviors, while therapeutic intervention mitigated established pain behaviors	[174]
	Preclinical (mouse)	CuMVttNGF vaccine	NGF vaccine alleviated spontaneous pain behavior in surgically induced OA	[175]
	Preclinical (rat)	Anti-NGF mAb	The injection of anti-NGF antibodies reduced pain scores in OA rats, improving their weight-bearing performance; however, it did not alleviate allodynia	[176]
Diabetic polyneuropathy (DPN)	Human	Anti-NGF mAb (Fulranumab)	This study offered evidence that in DPN patients, fulranumab reduces pain scores	[177]
	Human	Anti-NGF mAb (Tanezumab)	Tanezumab provided effective pain reduction in DPN	[178]
	Preclinical (mouse)	Humanized anti-NGF mAb (huAb45)	huAb45, an antibody capable of neutralizing the interaction between NGF and its receptor TrkA, has demonstrated efficacy in alleviating NP associated with DPN	[179]
Chemotherapy-induced peripheral neuropathy (CIPN)	Preclinical (rat)	Humanized anti-NGF mAb (DS002)	In three rat models of CIPN (paclitaxel, cisplatin, and vincristine), subcutaneous administration of DS002 demonstrated a significant prophylactic effect	[180]
	Preclinical (rat)	GW441756 (selective TrkA antagonist)	TrkA activation by NGF triggered sensory neuritogenesis and nociceptor sensitization, which can be prevented by TrkA inhibition. GW441756 reduced cisplatin-induced TRPV1-related nociceptor sensitization and prevented NP caused by cisplatin	[160]

## Abbreviations

The following abbreviations are used in this manuscript:

Akt	AKT serine/threonine kinase
AMPA	α-amino-3-hydroxy-5-methyl-4-isoxazolepropionic acid
AP-1	Activator protein 1
ASIC	Acid-sensing ion channels
BDNF	Brain-derived neurotrophic factor
cAMP	Cyclic adenosine monophosphate
CCI	Chronic constriction injury
CCL2	C-C motif chemokine ligand 2
CGRP	Calcitonin gene-related peptide
CIPN	Chemotherapy-induced peripheral neuropathy
CNS	Central nervous system
CRD	Cysteine-rich domain
CRD1	Cysteine-rich domain 1
CRD2	Cysteine-rich domain 2
CRD3	Cysteine-rich domain 3
CRD4	Cysteine-rich domain 4
CRM	Cysteine-rich motif
CSF	Cerebrospinal fluid
CX3CL1	C-X3-C motif chemokine ligand 1
DPN	Diabetic peripheral neuropathy
DRG	Dorsal root ganglion
EAE	Experimental autoimmune encephalomyelitis
ECD	Extracellular domain
ERK	Extracellular signal-regulated kinase
ERK1/2	Extracellular signal-regulated kinases 1/2
FAP-1	Fas-associated phosphatase-1
GABA	Gamma-aminobutyric acid
HIV	Human immunodeficiency virus
HSAN V	Hereditary sensory neuropathy type V
IASP	International Association for the Study of Pain
IgG	Immunoglobulin G
IL-1β	Interleukin 1 beta
IL-6	Interleukin 6
JNK	c-Jun N-terminal kinase
LBP	Low back pain
*l*-CDL	Levo-corydalmine
LRM	Leucine-rich motif
LTP	Long-term potentiation
mAb	Monoclonal antibody
MAPK	Mitogen-activated protein kinase
MAPKK	Mitogen-activated protein kinase kinase
MEK1/2	Mitogen-activated protein kinase kinase 1/2
mGluR	Metabotropic glutamate receptors
MMP9	Matrix metalloproteinase-9
mRNA	Ribonucleic acid
MS	Multiple sclerosis
NADE	p75^NTR^-associated cell death executor
Nav1.7	Voltage-gated sodium channel, type 1.7
Nav1.8	Voltage-gated sodium channel, type 1.8
Nav1.9	Voltage-gated sodium channel, type 1.9
NF-κB	Nuclear factor kappa-light-chain-enhancer of activated B cells
NGF	Nerve growth factor
NMDA	N-methyl-D-aspartate
NMR	Nuclear magnetic resonance
NO	Nitric oxide
NP	Neuropathic pain
NRAGE	Neurotrophin receptor-interacting MAGE protein
NRIF	Neurotrophin receptor-interacting factor
NT-3	Neurotrophin 3
NT-4	Neurotrophin 4
OA	Osteoarthritis
p65	NF-kB subunit p65
p75^ICD^	p75 intracellular domain
p75^NTR^	p75 neurotrophin receptor
pAb	Polyclonal antibody
PI3K	Phosphoinositide 3-kinase
PKA	Protein kinase A
PKB	Protein kinase B
PKC	Protein kinase C
PLCγ	Phospholipase C gamma
PNS	Peripheral nervous system
proNGF	Pro-nerve growth factor
Raf	Rapidly accelerated fibrosarcoma
Ras	Rat sarcoma virus protein
RIP2	Receptor-interacting protein kinase 2
RN	Red nucleus
SAXS	Small-angle X-ray scattering
SCN	Sciatic nerve cryoneurolysis
SNI	Spared nerve injury
SNRI	Serotonin–norepinephrine reuptake inhibitor
SNS	Somatic nervous system
Sp1	Specificity protein 1
TAK1	TGF-β-activated kinase 1
TCA	Tricyclic antidepressant
TN	Trigeminal neuralgia
TNFR	Tumor necrosis factor receptor
TNF-α	Tumor necrosis factor alpha
tPA	Tissue plasminogen activator
TRAF6	TNF receptor-associated factor 6
Trk	Tropomyosin receptor kinase
TrkA	Tropomyosin receptor kinase A
TRP	Transient receptor potential
TRPA1	Transient receptor potential ankyrin 1
TRPV1	Transient receptor potential vanilloid 1
UK	United Kingdom
VGCC	Voltage-gated calcium channel
VGSC	Voltage-gated sodium channel
